# Household air pollution disparities between socioeconomic groups in Chicago

**DOI:** 10.1088/2515-7620/ad6d3f

**Published:** 2024-09-03

**Authors:** William Isaac Krakowka, Jiajun Luo, Andrew Craver, Jayant M Pinto, Habibul Ahsan, Christopher S Olopade, Briseis Aschebrook-Kilfoy

**Affiliations:** 1 Institute for Population and Precision Health, the University of Chicago Biological Sciences Division, Chicago, United States of America; 2 Department of Public Health Sciences, the University of Chicago Biological Sciences Division, Chicago, United States of America; 3 Department of Surgery, Pritzker School of Medicine, the University of Chicago Biological Sciences Division, Chicago, United States of America; 4 Departments of Family Medicine and Medicine, Pritzker School of Medicine, the University of Chicago Biological Sciences Division, Chicago, United States of America

**Keywords:** PM, pollution, exposure, urbanicity, indoor environment

## Abstract

*Purpose*: To assess household air pollution levels in urban Chicago households and examine how socioeconomic factors influence these levels. *Methods*: We deployed wireless air monitoring devices to 244 households in a diverse population in Chicago to continuously record household fine particulate matter (PM_2.5_) concentration. We calculated hourly average PM_2.5_ concentration in a 24-hour cycle. Four factors—race, household income, area deprivation, and exposure to smoking—were considered in this study. *Results*: A total of 93085 h of exposure data were recorded. The average household PM_2.5_ concentration was 43.8 μg m^−3^. We observed a significant difference in the average household PM_2.5_ concentrations between Black/African American and non-Black/African American households (46.3 versus 31.6 μg m^−3^), between high-income and low-income households (18.2 versus 52.5 μg m^−3^), and between smoking and non-smoking households (69.7 versus 29.0 μg m^−3^). However, no significant difference was observed between households in less and more deprived areas (43.7 versus 43.0 μg m^−3^). *Implications*: Household air pollution levels in Chicago households are much higher than the recommended level, challenging the hypothesis that household air quality is adequate for populations in high income nations. Our results indicate that it is the personal characteristics of participants, rather than the macro environments, that lead to observed differences in household air pollution.

## Introduction

Air pollution ranks among the leading causes of premature death globally. In 2018, the World Health Organization (WHO) reported that an estimated 7 million deaths are attributable to the combined effects of indoor and ambient air pollution [1]. Notably, previous research has identified ambient particulate matter (PM) exposure as a determinant of increased all-cause mortality or disability-adjusted life years [2–5], with PM_2.5_ having been specifically shown to increase the risk for pulmonary, cardiovascular and neurological diseases [6–9]. In 2021, the WHO released new guidelines detailing health effects and standard levels of air pollution for both outdoor and indoor environments [1]. Regarding outdoor and ground-level air pollution, analytical infrastructures used to measure ambient air pollution are sophisticated, with pixelated PM_2.5_ levels being widely available at 0.01° × 0.01° geographical areas [10–12]. Despite how advanced these ambient PM_2.5_ collection methods are, there remain gaps in data accessibility for household environments, limiting our understanding of the impacts of household air pollution and associated health effects. The scarcity of data on indoor environments is particularly striking when considering that urban populations spend up to 90% of their time indoors [13, 14]. However, recent development in low-cost air quality sensors offer a cost-effective approach to researching the indoor environment on a large scale, contributing to the growing body of data on household air pollution [5, 15–17].

Previous research has highlighted exposure disparities in outdoor environments, showing that socioeconomically disadvantaged groups face higher levels of ambient air pollution [18–20]. However, the degree to which these disparities extend to indoor environments remains underexplored, especially among Black and African American households. Since outdoor environments at least partially contribute to indoor environments [21–23], we hypothesize that these exposure levels may be impacted by race, contextual deprivation, household income, and smoking status. Race, deprivation, and household income are all adverse exposures to pollutants in neighborhoods housing minority populations where pollution is prevalent [24–26], and these exposures are mediated by neighborhood-level characteristics of deprivation and household income [27–34]. Conversely, smoking actively contributes PM to indoor environments, so its inclusion is necessary in measuring potential confounding that may arise from smoking behavior [35–37].

Given the nature and characteristics of built environments, evidence suggests that the levels of household air pollution often exceed the levels of ambient air pollution [38, 39]. However, the existing research on the relationship between household and ambient pollution levels remain incomplete [38], underscoring the need for increased scaling of household air quality data collection.

Within this context, we considered four socioeconomic variables in our study: race, household income, contextual deprivation, and smoking. These variables were chosen because prior research suggested their substantial impact on indoor air quality, and investigation of these variables is in accordance with the longitudinal cohort goals of the Chicago Multiethnic Prevention and Surveillance Study (COMPASS) that seeks to understand why certain racial or population sub-groups are high risk for a variety of chronic diseases [40]. Understanding how the health of Chicagoans is shaped by where they live is central to addressing these overarching cohort study aims, and our study provides preliminary data on household environments for certain racial and population sub-groups.

In this analysis, indoor air pollution refers to the aggregation of indoor environments where PM can accumulate, such as inside homes, at workplaces, or inside schools. Conversely, household air pollution—the target of this pilot research program—remains understudied when compared to aggregated indoor environments. We will investigate exclusively household environments.

The goal of this pilot study is to determine household air pollution exposure levels in a diverse study population in Chicago and elaborate on whether differences in socioeconomic factors contribute to exposure disparities in household air quality.

## Methods

This study leverages data from COMPASS, an ongoing, longitudinal cohort study with a focus on underrepresented populations on the South Side of Chicago [40]. The deidentified data that support the findings of this study are available from the corresponding author upon reasonable request. Since 2013, COMPASS has enrolled over 13,000 participants. Beginning in March 2019, COMPASS deployed low-cost air quality monitoring devices to participants to study the household environment. Distribution was generally paused for two years during the COVID-19 pandemic but has since resumed, totaling 244 sampled households. Distribution began in March of 2019 and continued consistently through February of 2020, with cohort data being gathered throughout each month of that first year of piloting. A handful of households were enrolled during December 2020, January 2021, and August 2021. Beginning in January 2022 and continuing through December 2023, distribution resumed for nearly all months during this two-year time span except November 2022 and February 2023. Three air monitoring devices were used, including Purpleair PA-II-SD (henceforth ‘Purpleair’, n = 117), Edimax Edigreen Home (henceforth ‘Edigreen’, n = 121), and Edimax AirBox (henceforth ‘Edimax’, n = 11). Five households received both Edigreen and Edimax devices. Field studies conducted by the Air Quality Sensor Performance Evaluation Center have quantified their sensitivity, with Purpleair (R^2^ = 0.93–0.97), Edigreen (R^2^ = 0.82–0.83), and Edimax (R^2^ = 0.61–0.87) showing reasonable predictive power when compared to EPA-approved air quality monitoring devices [41]. All R^2^ coefficients calculated the linearity between the sensor and available federal reference equipment or equivalent [41]. Deployment of different device types was part of piloting feasibility. These monitoring devices measured concentrations of PM_1.0_, PM_2.5_, and PM_10.0_. Results of all hourly PM concentrations can be found in Supplemental Materials.

Participants were asked to set up the devices on a table or shelf approximately 3 feet above the floor in the room of their home where their daily activities predominantly occur. The devices recorded PM concentrations continuously for the duration of their time in participants’ homes, with durations ranging from 1 day to multiple weeks. Devices were then retrieved by research assistants. We calculated the average hourly concentration of each PM type for each household. We stratified each household by the four selected socioeconomic factors, respectively, and described the trends of household air pollution in stratified groups. These factors were chosen as important predictors of socioeconomic status of study participants. Our COMPASS cohort has recruited study participants who are most representative of socioeconomically disadvantaged populations throughout greater Chicago, which helps us to draw conclusions about the city’s most disadvantaged population sub-groups, aligning with our COMPASS cohort expectations.

Despite best efforts to control where air monitoring devices are displayed in the household, we are unable to consider vertical variation as a potential confounder of collected data among our participant households. While it is true that there is a vertical component to PM suspension during certain times of the day or at certain apartment elevations [42–45], we are unable to standardize the height at which our participant households placed their air quality monitor. To control variability to the best of our ability, our instructions to participants remained uniform, so all participants received the same information about where they should be placing their air quality monitor.

To contrast household environments with outdoor environments, we leveraged raster data from Washington University-St. Louis. These fine-resolution raster images were used to generate mean concentrations of ground-level PM at each participant household. Raster images offer summarized ground-level PM for all households at a 0.01° × 0.01° geographic area, providing descriptive data to contrast household environments from outdoor environments. However, satellite imagery data is only available by month through 2022. To compensate for the lack of existing data for 2023, we made the assumption that ground-level PM did not considerably vary by month, year over year. So, for households reporting data in 2023, we used satellite data from 2022 to develop their outdoor exposure levels. Between 2019 and 2022, satellite PM levels varied between 6.0—13.6 μg m^−3^ [10]. In the event that household data collection spans multiple months, each month of active data collection was mapped to corresponding raster imagery, and the resulting ground-level concentrations were averaged for all valid months of active data collection for a household.

We calculated the uncertainty of our summary statistics by considering both intra- and inter-household variations. All analyses were conducted using R (version 4.3.0) [46] for statistical analysis or ArcGIS Online [47] for ecological analysis of ground-level PM. All participants provided written informed consent to participate in this study.

## Results

A total of 244 households were sampled for household air quality monitoring. These households recorded valid PM_2.5_ data for a total of 93,085 h (381.5 h per household on average).

**Figure 1.**
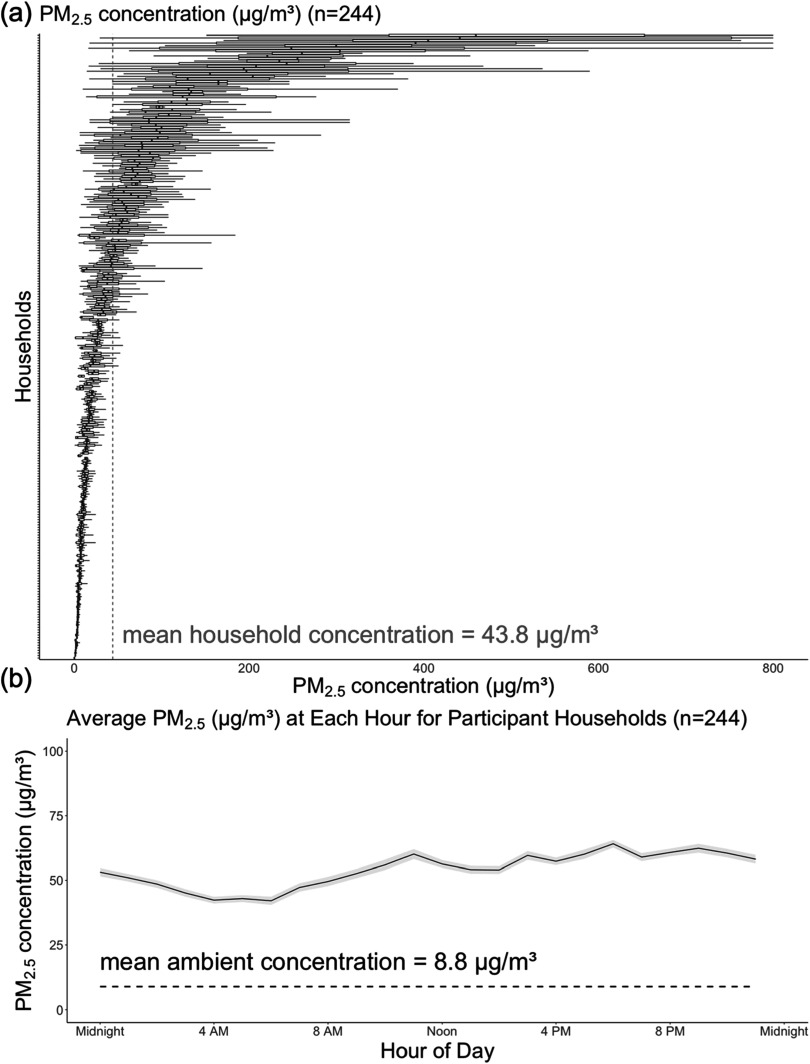
Average particulate matter readings and interquartile ranges for all PM_2.5_ data. The marked vertical red line indicates the daily average particulate matter reading concentration (μg/m^3^). The marked horizontal black line indicates the mean ambient PM_2.5_ for all households.

The WHO guidelines on household air pollution recommend that mean PM_2.5_ concentrations not exceed an average exposure of 5.0 μg m^−3^ per year [1], while also recommending that mean PM_2.5_ concentrations not exceed concentrations of 15.0 μg m^−3^ more than 3–4 days per year [1]. Our results show average PM concentrations that far exceed the recommended levels for PM_2.5_ (figure 1), with an average level of 43.8 μg m^−3^. Only 21 households met the WHO recommendation on average exposure for PM_2.5_ (figure 1).

**Figure 2.**
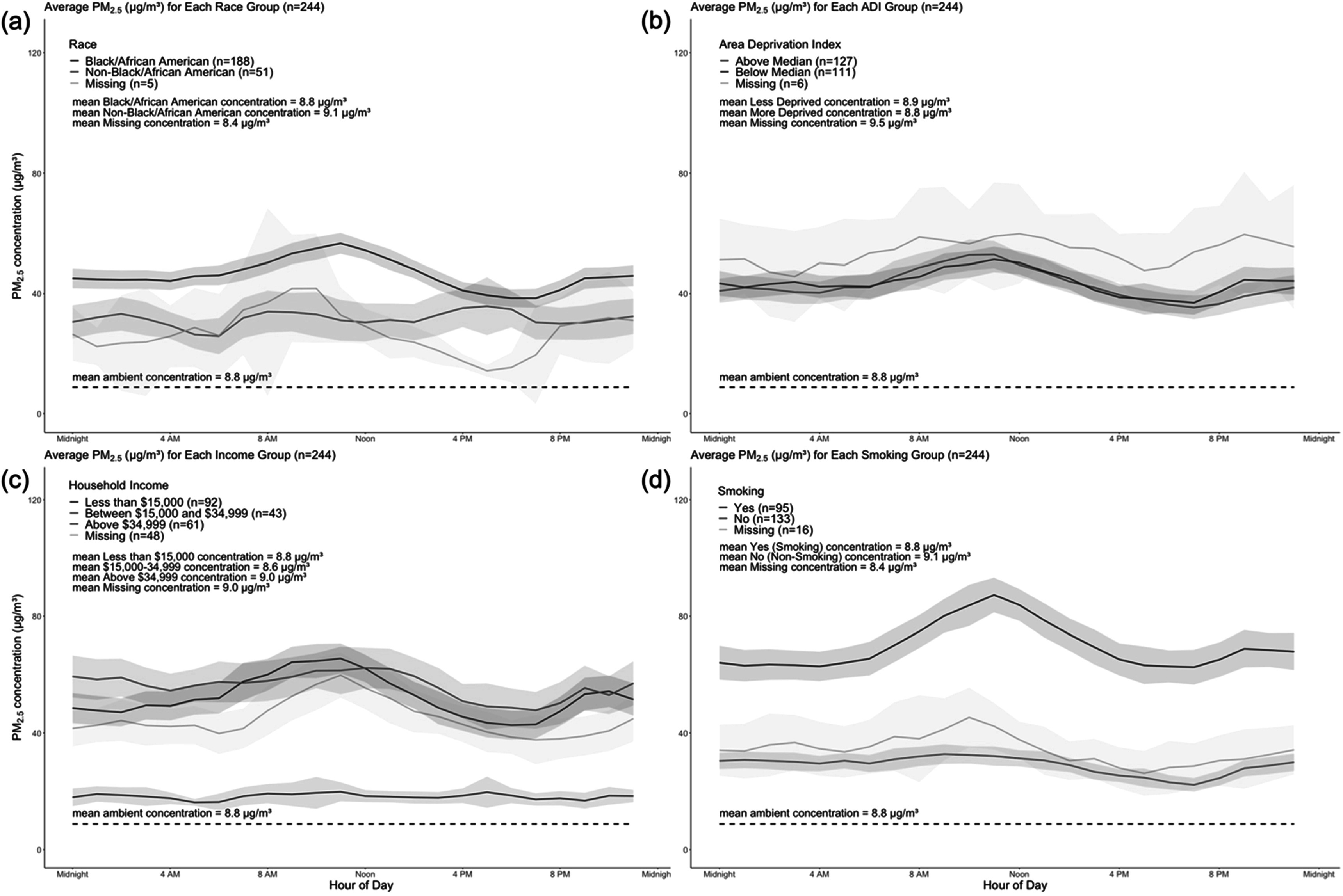
Average PM_2.5_ concentrations (μg/m^3^) for all households, stratified by Race (a), Area Deprivation Index (b), Household Income (c), and Smoking Status (d). Figure A describes differences in Black/African American and non-Black/African American households. Figure B describes differences between households above the median for reported Area Deprivation Index values (more disadvantaged), and below the median for reported Area Depravation Index values (less disadvantaged). Figure C describes the differences between different household income bins, including < $15,000, $15,000-$34,999, and > $34,999. Figure D describes differences between households with a smoker in the preceding 12 months and/or if the resident is a current smoker (Yes) and those who have not and are not a current smoker (No). All figures include a visualization for missing self-reported data from COMPASS data collection. All figures provide summary data for ambient PM, stratified by grouping variables. The marked horizontal black line indicates the average ambient PM_2.5_ for all households for comparison.

In a 24-hour period, we observed an increasing trend in PM_2.5_ levels in the morning, leading to a peak during the same time period. This could be due to elevated traffic levels or personal household behaviors like smoking, which we observed in households exposed to smoking and did not observe in households not exposed to smoking (figure 2) [38, 39, 47]. After the morning, the observed concentrations decrease during the afternoon, but again increased during the early evening. This source of increase may be due to human activities like cooking or elevated traffic during rush hour [48]. We finally observed a decreasing trend in the very early morning after midnight and before 5 AM. Our data suggest that the household air pollution levels are far higher than the recommended levels, and these levels of exposure vary by socioeconomic variables (table 1).

**Table 1.** Average and standard deviation observations of PM_2.5_ concentrations, stratified by grouping variables. average ground-level PM_2.5_, stratified by grouping variables, is provided for comparison.

	All households	Race		Area depravation		Household income			Smoking status	
		Black/African American	Non-black/African American	Less deprived	More deprived	Below $15,000	Between $15,000 and $34,999	Above $34,999	Yes	No
Sample Size	n = 244	n = 188	n = 51	n = 127	n = 111	n = 92	n = 43	n = 61	n = 95	n = 133
Total number of hours recorded	93,085	77,538	13,733	44,770	44,363	33,758	22,324	22,956	33,087	53,857
Average (μg/m^3^)	43.8	46.3	31.6	43.7	43.0	52.5	56.3	18.2	69.7	29.0
Intra-household standard deviation	19.2	18.1	23.2	19.1	19.7	19.8	18.6	13.4	21.8	15.3
Inter-household standard deviation	22.8	22.9	22.6	22.0	23.9	25.6	22.9	12.1	28.9	17.1
Overall standard deviation	29.8	29.2	32.4	29.1	31.0	32.4	29.5	18.1	36.2	22.9
Average ambient PM_2.5_	8.8	8.8	9.1	8.9	8.8	8.8	8.6	9.0	8.8	8.9

Notably, our data illustrate significant differences in hourly PM_2.5_ concentrations between Black/African American and Non-Black/African American participants for most hours in a 24-hour period (figure 2(a)**,** table 1). The average difference in PM_2.5_ concentration is 14.7 μg m^−3^ between these two groups (46.3 μg m^−3^ in Black/African American group versus 31.6 μg/m^3^ in non-Black/African American group). The difference appears highest around noon, with the smallest difference occurring during the evening. These differences between Black/African American and Non-Black/African American participants expand again at night and in the early morning. For example, peak concentrations of PM_2.5_ in the morning were 56.6 μg m^−3^ and 34.0 μg m^−3^ for Black/African American and Non-Black/African American households, respectively. The difference in PM_2.5_ reduced in the early evening, with a concentration of 38.5 μg m^−3^ and 34.8 μg m^−3^ at 6 PM for these groups.

Similar to race, our data show significant differences between household income groups. Household air pollution concentrations remained stable and low in households with income > $34,999, with an average level of 18.2 μg m^−3^ for PM_2.5_. In comparison, we observed higher average and substantial fluctuations for households with lower income: over a 24-hour period, we observed an increasing trend in the morning, peaking around noon; then a decreasing trend in the afternoon, followed by a slight increase at night. Among households with reported incomes below $15,000, the peak concentration of PM_2.5_ was 51.9 μg m^−3^ in the morning, increasing to 65.6 μg m^−3^ in the afternoon, and ultimately decreasing to 42.7 μg m^−3^ at night. During those same hours, households earning over $34,999 per year reported concentrations of 16.2 μg m^−3^ , 19.8 μg m^−3^, and 18.5 μg m^−3^.

Our data show significant differences between smoking groups. Individuals were classified into the smoking group if they self-reported current smoking and/or if they self-reported that an individual (including themselves) had smoked inside their household in the preceding 12 months. Household air pollution levels remained relatively stable and similar among households that reported they did not smoke and among households with missing smoking data (figure 2). Among households with smoking data, we observed relatively stable concentrations during the early morning (63.3 μg m^−3^ at 3 AM), observed a large increase around the afternoon (87.3 μg m^−3^ at 11 AM), and observed a dip in PM_2.5_ concentrations in the early evening (62.6 at μg/m^3^ at 7 PM). Additionally, the average value of households who do not smoke was the second smallest average PM reading we detected, at 29.0 μg m^−3^ (outside of households earning above $34,999 per year), while smoking households reported the clear highest average PM reading (69.7 μg m^−3^).

In contrast with race, smoking status, and household income, the differences between area deprivation groups were small. The average PM_2.5_ concentrations are 43.7 μg m^−3^ and 43.0 μg m^−3^ in the less deprived and more deprived groups, respectively. No significant difference is observed between the two groups. Overall, compared to more individual-level race, household income, and smoking status variables, the area deprivation at the contextual level demonstrates a minor role in the household exposure disparities.

The exposure disparities between socioeconomic groups remained when we exclude households that reported smoking (Supplemental tables S13-S21; figure S4), when we exclude households with average PM_2.5_ readings below 200 μg m^−3^ (figure S3), and when we exclude households with data spanning fewer than 3 days (figure S5). Results for PM_1.0_ and PM_10.0_ demonstrate consistent patterns and can be found in Supplemental Materials (Supplemental tables S1-S12; figures S1, S2). Additional consideration of seasonality may be found in the Supplemental Materials. Seasonality considers the potential that gas stoves are used to heat homes during colder months, of which we observed mild increases in concentrations of particulate matter of all concentrations in the evenings during winter months (figures S6–S8). We stratified the seasonality analysis by household income, and the trends of household income persisted when stratifying by season, except for during the winter. All households appeared to follow the same pattern of increased particulate matter exposure in evenings.

## Discussion

As a pilot study leveraging our diverse cohort in Chicago, Illinois, our research contributes to the growing pool of data on household air pollution levels and also suggests a relationship between socioeconomic factors. To frame our data against other similar global findings, research in French homes revealed a similar relationship between socioeconomic factors and household air pollutants, where increasing income levels were generally associated with lower average PM_2.5_ concentrations, with households in the highest income group reporting the lowest mean household concentrations [49]. For the highest income population, the French reported an average PM_2.5_ level of 37.2 μg m^−3^ [49], which is higher than what we observed in our study. From a global perspective, these concentration levels in high income countries (HIC) are comparable to those in low- and middle-income countries (LMIC). Recent findings show that a microenvironment involving cooking and eating ranged from reported household PM_2.5_ levels of 39.9–427.5 μg m^−3^ in LIMCs, such as Pakistan, Bangladesh, and Cambodia [50]. Our data are also comparable to household PM_2.5_ levels reported during cooking and eating in Malaysia (39.9 μg m^−3^) and Thailand (58.2 μg m^−3^), with some of our sampled households far exceeding these reported national averages [50]. The general consensus is that household air pollution is a more concerning public health issue in LMIC than in HIC because of clean technology and less use of biomass fuel; however, our monitoring data challenges this consensus [39, 51, 52]. These results help to show that the burden of adverse PM exposure remains worrisome for the US, especially among socioeconomically disadvantaged populations [49].

Our selected variables were chosen to compare understood relationships between indoor environments and population sub-groups of interest. Neighborhood characteristics and surrounding environments of predominantly Black and African American communities is often the designated mechanism for adverse PM exposure for Black and African American individuals. Chicago is among the most segregated cities in the United States [53], with unequal health disparities on the city’s South Side [53]. As one of the nation’s largest contiguous African American Communities, Chicago’s South Side faces disproportionate environmental burdens in conjunction with its demonstrated disparities in health outcomes [20, 54–56]. Notably, there exist racial and socioeconomic disparities in living in proximity to polluting industrial facilities, as Black individuals at lower education levels and income levels were significantly more likely to live within a mile of a polluting facility [56], and air quality in Chicago among marginalized communities is threatened by the expansion of polluting industries [57]. Recent investigation into potential risk factors stratified by race have found that Black and African American individuals are exposed to higher-than-average concentrations from all polluting sectors, including industrial, vehicular, construction, and commercial cooking-based PM_2.5_ [32]. From a structural perspective, environmental racism has aggregated Black and African American individuals into urban regions that experience disproportionate exposure burdens from pollution sources. Urban histories of redlining, among other racial histories intended to localize minority residency, have contributed to creating environmental injustice and physical environments where PM_2.5_ is unavoidable for minorities [25, 57, 58]. As particulate matter from surrounding sources may be a contributor to indoor environments [35], these risk factors help to characterize the South Side’s exposure to particulate matter and environmental toxins, and this research analyzes household environments against the backdrop of these multiple risk factors. For these reasons, and in conjunction with previous research on this particular cohort, we identify race as a potential risk factor for worsened indoor PM exposure, which has been a relatively understudied risk factor when compared to other chosen socioeconomic variables [59].

Consideration of Area Depravation Index (ADI) would help to elucidate the contributions of the surrounding environment on household environments. The Area Depravation Index systematically ranks neighborhoods based on socioeconomic disadvantage, and it considers income, education, employment, and housing quality [60, 61]. Given our hypotheses about historically Black neighborhoods and their interaction with environmental PM, including ADI helps to understand how neighborhood characteristics outside of race contribute to household environments. Notably, reduced housing quality may serve as a risk factor for household PM exposure, as lower quality households may inefficiently exchange household and outdoor air. More generally, ADI considers neighborhood characteristics as the mechanism to adverse air quality exposures. Using ADI, we are able to approximate neighborhood characteristics for our participant households to understand the neighborhood-level contribution to adverse particulate matter exposure, and we contribute to existing research using area depravation as a proxy for socioeconomic status of study participants [62–66].

Household income was considered as a more participant-specific descriptor of socioeconomic standing. Our hypothesized mechanism for improved socioeconomic standing would suggest that higher household incomes may afford individuals the opportunity to personally reduce their risk factors for PM exposure. These personal reductions may be via improved housing quality, improved air exchange mechanisms, or higher quality cookware and household appliances. However, these aforementioned personal reductions are not exhaustive, so grouping individuals by their income level will help us to understand how adverse air quality affects low-income households. Notably, our grouping of households allows us to understand how a household earning less than $15,000 per year, the federal poverty line designated for a one-person household [67], may be impacted by adverse household air quality, which parallels existing studies using household income to define socioeconomic status [49, 62, 68].

Lastly, smoking status was included as a potential risk factor to our dataset, as the mechanism by which tobacco smoke deposits PM_2.5_ into trapped, indoor spaces is well understood [35–37]. If tobacco smoke is unable to escape indoor environments, it disproportionately contributes to worsened PM levels. We hope to further understand how indoor smoking behavior contributes to household environments. In the event that tobacco smoking confounded our data results, we have included an analysis of non-smoking households exclusively in the Supplemental Materials.

Within this analysis, we are able to parallel the investigation of sparse, yet existing literature on adverse air quality exposure among low-income and smoking population sub-groups [49, 62, 68–70]. In addition to this research, we are able to magnify these results to the Chicagoland geography and apply our novel findings to understudied relationships between indoor air pollution exposure and race [59]. Since our results do not show robust significance when comparing area deprivation groups, this may suggest that household air pollution exposure may be more attributable to individual factors instead of neighborhood exposures, such as individual behaviors and smoking status. When considering individual race, household income, and smoking status, these metrics appear to contribute much more to the household air quality than a household’s neighborhood surroundings.

Prior studies have mentioned that Black and African American individuals live in more deprived areas with higher air pollution [25, 57, 58], but little is known how this is reflected in indoor environment. Our study provides opportunities to examine how outdoor exposure inequalities would influence the indoor environment. While we have identified industrial and environmental sources of pollution as potential contributors to adverse indoor environments [25, 32, 35, 56–58], our observations suggest that macro environments (neighborhood ADI and ambient PM_2.5_) did not predict the difference in household air pollution level (table 1). By contrast, it is the personal characteristics, such as smoking, household income, and race, that lead to difference in indoor environment. These personal contributions may be mediated by our observed socioeconomic variables of interest, as previous research demonstrated adverse exposures to smoking behaviors among Black and African American populations in Chicago [71] and has shown how household income and socioeconomic status may mediate usage of gas stoves for cooking and heating of low-income households [72–75]. Our findings offer novel insights about environmental exposure disparities.

Two unstudied variables of interest, household educational attainment and aforementioned household cooking behavior, should be considered in future analyses. Since our findings illustrate that particulate matter concentrations remain high in the mornings when no cooking would be happening, this may suggest inefficient air exchange among participant households. In 2023, the CDC recommended 5+ indoor air exchanges for optimal reduction of individual indoor exposures during the COVID-19 pandemic [76]. Additionally, CDC research on the relationship between indoor PM_2.5_ and air exchange rates found that buildings where quarterly air exchange rates above the 25th percentile (0.31/hour) reported lower PM_2.5_ concentrations than buildings with exchange rates below the 25th percentile [77]. In comparing expected concentrations of PM_2.5_ during working hours, these researchers found that expected PM_2.5_ was 30% lower in second quartile buildings (0.31–0.47/hour) when compared to first quartile buildings (< 0.31/hour), 29.3% lower in third quartile buildings (0.47–0.84/hour), and 13.6% lower in fourth quartile buildings (> 0.84/hour) [77]. These data suggest that even modest improvements to air exchange reduce expected indoor PM_2.5_. Given these findings, our data suggest that many participant households may have an inadequate exchange rate of indoor air, especially among low income and smoking households. Conversely, we observe very stable particulate matter concentrations among high income and non-smoking populations. These variables may correlate to the efficiency of air exchange within a household. Our findings warrant future research into the sufficiency of hourly air exchanges among an urban population. Given our findings that show variable PM exposure in the evenings, the relationship of smoking behavior during the day and household educational levels may reveal insufficient hourly air exchanges in the household. Future research should consider the interactive effects of individual behaviors like smoking and cooking and the efficiency of intra-household hourly air exchanges.

Our analysis relied on accurate and continued data collection for PM readings during our study period. Our study has limitations from the style and scope of the study design. First, our relatively small sample size—both in total households and in some selected demographics—restricts the generalizability of our data over a large geographic area. Second, given that COMPASS is a predominantly Black and African American cohort study, we are unable to extend our results to demographic groups beyond Black and African American participants. Third, although our sample measures multiple PM concentrations, we lack data on accurate placement of air quality monitoring devices within the household, which may affect the readings that low-cost air quality monitoring devices report. Fourth, we relied on multiple device types, as this was part of piloting feasibility. Fifth, we did not consider household infrastructure, such as housing quality, ventilation, air exchange, and presence of other household toxicants beyond ADI. Lastly, our study was conducted in an urban setting, and our findings may not apply to rural areas. Despite these limitations, this study adds to the growing body of data and literature describing the health risks of household air pollution. Our findings emphasize the importance of considering socioeconomic factors and disparities in future investigations of household air quality and the need for continued targeted intervention work and community partnerships in disadvantaged populations, especially in urban settings where household and ambient air pollution levels may be highest.

## Conclusion

Our results, together with recent studies, corroborate the utility of low-cost sensors in collecting household air quality household data over large populations [5, 78]. Future research would benefit from continued use of these devices to investigate the relationship between socioeconomic disadvantage and adverse household air quality exposure. Given the widely understood health risks associated with PM exposure, monitoring the household environment is important for reducing incidence of chronic respiratory conditions and all-cause mortality from PM exposure. In settings where populations are spending the majority of time indoors—especially given the increasing propensity of working indoors or from at-home settings [5]—addressing disparities in household environments remains crucial. Even in cities in HIC, the famous ‘Harvard Six Cities Study’ showed the burden of fine particulate matter exposure, with individuals being at an increased risk of developing lung cancer and cardiovascular diseases at similar levels of exposure to what we have found in our study [79]. Based on our data, we believe this pilot research study describes how household environments for a predominantly Black and African American cohort in Chicago are at unhealthy levels of exposure when compared to WHO recommendations [1], and our findings suggest that personal contributions to household air pollution may contribute more to indoor environments than environmental sources of air pollution.

## Data Availability

The data cannot be made publicly available upon publication because they contain sensitive personal information. The data that support the findings of this study are available upon reasonable request from the authors.

## Supporting information

Additional PM_1.0_, PM_10.0_ and sensitivity analyses, including exclusion of households with low PM_2.5_ concentration (< 200 μg m^−3^), households exposed to smoking, and households with readings fewer than 3 days are found in Supplemental Materials. Analysis of seasonality, stratified by household income level, is found in Supplemental Materials.

## Funding

The author(s) disclosed receipt of the following financial support for the research, authorship, and/or publication of this article: This work was supported by the funding from NIH grants P30ES027792 and 1OT2OD036445.

## Disclosure

The author(s) declared no potential conflicts of interest with respect to the research, authorship, and/or publication of this article.

## References

[1] World Health Organization (2021). WHO Global air Quality Guidelines: Particulate Matter (PM2.5 and PM10), Ozone, Nitrogen Dioxide, Sulfur Dioxide and Carbon Monoxide.

[2] Franklin M, Zeka A, Schwartz J (2007). Association between PM2.5 and all-cause and specific-cause mortality in 27 US communities. J Expo Sci Environ Epidemiol.

[3] Han C (2022). Mortality burden due to long-term exposure to ambient PM2.5 above the new WHO air quality guideline based on 296 cities in China. Environ. Int..

[4] Forouzanfar M H (2016). Global, regional, and national comparative risk assessment of 79 behavioural, environmental and occupational, and metabolic risks or clusters of risks, 1990–2015: a systematic analysis for the global burden of disease study 2015. Lancet.

[5] Bousiotis D, Alconcel L N S, Beddows D C S, Harrison R M, Pope F D (2023). Monitoring and apportioning sources of indoor air quality using low-cost particulate matter sensors. Environ. Int..

[6] Xia X, Zhang A, Liang S, Qi Q, Jiang L, Ye Y (2017). The association between air pollution and population health risk for respiratory infection: a case study of shenzhen, China. Int. J. Environ Res. Public Health.

[7] Dominici F (2006). Fine particulate air pollution and hospital admission for cardiovascular and respiratory diseases. JAMA.

[8] Fu J, Jiang D, Lin G, Liu K, Wang Q (2015). An ecological analysis of PM _2.5_ concentrations and lung cancer mortality rates in China. BMJ Open.

[9] Cristaldi A (2022). Possible association between PM2.5 and neurodegenerative diseases: a systematic review. Environ. Res..

[10] Washington University-St. Louis (2024). https://sites.wustl.edu/acag/datasets/surface-pm2-5/.

[11] Shiferaw A B, Kumie A, Tefera W, Giri B (2023). The spatial and temporal variation of fine particulate matter pollution in Ethiopia: data from the atmospheric composition analysis group (1998–2019). PLoS One.

[12] Rojas N Y (2023). Road transport exhaust emissions in Colombia. 1990–2020 trends and spatial disaggregation. Transp Res Part Transp Environ..

[13] Doyi I N Y, Strezov V, Isley C F, Yazdanparast T, Taylor M P (2020). The relevance of particle size distribution and bioaccessibility on human health risk assessment for trace elements measured in indoor dust. Sci. Total Environ..

[14] Monn C (1997). Particulate matter less than 10 μm (PM10) and fine particles less than 2.5 μm (PM2.5): relationships between indoor, outdoor and personal concentrations. Sci. Total Environ..

[15] Jovašević-Stojanović M, Bartonova A, Topalović D, Lazović I, Pokrić B, Ristovski Z (2015). On the use of small and cheaper sensors and devices for indicative citizen-based monitoring of respirable particulate matter. Environ. Pollut..

[16] Yang C T, Chen H W, Chang E J, Kristiani E, Nguyen K L P, Chang J S (2021). Current advances and future challenges of AIoT applications in particulate matters (PM) monitoring and control. J. Hazard. Mater..

[17] Sá J P, Alvim-Ferraz M C M, Martins F G, Sousa S I V (2022). Application of the low-cost sensing technology for indoor air quality monitoring: a review. Environ Technol Innov..

[18] Pratt G, Vadali M, Kvale D, Ellickson K (2015). Traffic, air pollution, minority and socio-economic status: addressing inequities in exposure and risk. Int. J. Environ Res. Public Health..

[19] Houston D, Wu J, Ong P, Winer A (2004). Structural disparities of urban traffic in southern california: implications for vehicle-related air pollution exposure in minority and high-poverty neighborhoods. J. Urban Aff..

[20] Jones M R (2014). Race/ethnicity, residential segregation, and exposure to ambient air pollution: the multi-ethnic study of atherosclerosis (MESA). Am. J. Public Health.

[21] Xie Y, Zhao B (2018). Chemical composition of outdoor and indoor PM _2.5_ collected during haze events: transformations and modified source contributions resulting from outdoor-to-indoor transport. Indoor Air.

[22] Ji W, Zhao B (2015). Contribution of outdoor-originating particles, indoor-emitted particles and indoor secondary organic aerosol (SOA) to residential indoor PM2.5 concentration: a model-based estimation. Build. Environ..

[23] Meng Q Y (2005). Influence of ambient (outdoor) sources on residential indoor and personal PM2.5 concentrations: analyses of RIOPA data. J Expo Sci Environ Epidemiol..

[24] Woo B, Kravitz-Wirtz N, Sass V, Crowder K, Teixeira S, Takeuchi D T (2019). Residential segregation and racial/ethnic disparities in ambient air pollution. Race Soc. Probl..

[25] Motairek I, Chen Z, Makhlouf M H E, Rajagopalan S, Al-Kindi S (2023). Historical neighbourhood redlining and contemporary environmental racism. Local Environ..

[26] Lane H M, Morello-Frosch R, Marshall J D, Apte J S (2022). Historical redlining is associated with present-day air pollution disparities in U.S. cities. Environ. Sci. Technol. Lett..

[27] Milojevic A (2017). Socioeconomic and urban-rural differentials in exposure to air pollution and mortality burden in England. Environ Health..

[28] Næss Ø, Piro F N, Nafstad P, Smith G D, Leyland A H (2007). Air pollution, social deprivation, and mortality: a multilevel cohort study. Epidemiology.

[29] Wong C M (2008). The effects of air pollution on mortality in socially deprived urban areas in Hong Kong, China. Environ. Health Perspect..

[30] Bowe B, Xie Y, Yan Y, Al-Aly Z (2019). Burden of cause-specific mortality associated with PM _2.5_ air pollution in the United States. JAMA Netw Open.

[31] Jbaily A (2022). Air pollution exposure disparities across US population and income groups. Nature.

[32] Tessum C W, Paolella D A, Chambliss S E, Apte J S, Hill J D, Marshall J D (2021). PM _2.5_ polluters disproportionately and systemically affect people of color in the United States. Sci. Adv..

[33] Mikati I, Benson A F, Luben T J, Sacks J D, Richmond-Bryant J (2018). Disparities in distribution of particulate matter emission sources by race and poverty status. Am J Public Health.

[34] Kioumourtzoglou M A, Schwartz J, James P, Dominici F, Zanobetti A (2015). PM2.5and mortality in 207 US cities: modification by temperature and city characteristics. Epidemiology.

[35] Environmental Protection Agency (2024). Sources of Indoor Particulate Matter (PM) [Internet]. USEPA.

[36] Ni Y, Shi G, Qu J (2020). Indoor PM2.5, tobacco smoking and chronic lung diseases: a narrative review. Environ. Res..

[37] Semple S, Apsley A, Azmina Ibrahim T, Turner S W, Cherrie J W (2015). Fine particulate matter concentrations in smoking households: just how much secondhand smoke do you breathe in if you live with a smoker who smokes indoors?. Tob Control..

[38] Leung D Y C (2015). Outdoor-indoor air pollution in urban environment: challenges and opportunity. Front Environ Sci [Internet].

[39] Zhang J (J ), Smith K R (2003). Indoor air pollution: a global health concern. Br. Med. Bull..

[40] Aschebrook-Kilfoy B (2020). Cohort profile: the Chicago multiethnic prevention and surveillance study (COMPASS). BMJ Open.

[41] South Coast Air Quality Management District (2024). PM sensor evaluations [Internet]. Air Quality Sensor Performance Evaluation Center.

[42] Ainiwaer S (2022). Characterization of the vertical variation in indoor PM2.5 in an urban apartment in China. Environ. Pollut..

[43] Liao H, Lai Y, Chao H J, Wu C (2023). Vertical characteristics of potential PM2.5 sources in the Urban environment. Aerosol Air Qual. Res..

[44] Jung K H (2011). Vertical gradients of residential indoor and outdoor polycyclic aromatic hydrocarbons, black carbon, particulate matter in New York City. Epidemiology.

[45] Zheng S (2022). Vertically-resolved indoor measurements of air pollution during Chinese cooking. Environ Sci Ecotechnology.

[46] RStudio Team (2022). RStudio: integrated development environment for R. RStudio v4.3.0. [Internet]. RStudio PBC.

[47] Tong Z, Chen Y, Malkawi A, Adamkiewicz G, Spengler J D (2016). Quantifying the impact of traffic-related air pollution on the indoor air quality of a naturally ventilated building. Environ. Int..

[48] Shehab M, Pope F D, Delgado-Saborit J M (2021). The contribution of cooking appliances and residential traffic proximity to aerosol personal exposure. J. Environ Health Sci. Eng..

[49] Brown T (2015). Relationships between socioeconomic and lifestyle factors and indoor air quality in French dwellings. Environ. Res..

[50] Shimada Y, Matsuoka Y (2011). Analysis of indoor PM2.5 exposure in Asian countries using time use survey. Sci. Total Environ..

[51] Mannucci P, Franchini M (2017). Health effects of ambient air pollution in developing countries. Int. J. Environ Res. Public Health.

[52] Bruce N, Perez-Padilla R, Albalak R (2000). Indoor air pollution in developing countries: a major environmental and public health challenge. Bull World Health Organ..

[53] Orsi J M, Margellos-Anast H, Whitman S (2010). Black-white health disparities in the United States and Chicago: a 15-year progress analysis. Am. J. Public Health.

[54] Shanahan C E, Spak S N, Martinez A, Hornbuckle K C (2015). Inventory of PCBs in Chicago and opportunities for reduction in airborne emissions and human exposure. Environ. Sci. Technol..

[55] King K E (2015). Chicago residents’ perceptions of air quality: objective pollution, the built environment, and neighborhood stigma theory. Popul Environ..

[56] Mohai P, Lantz P M, Morenoff J, House J S, Mero R P (2009). Racial and socioeconomic disparities in residential proximity to polluting industrial facilities: evidence from the Americans’ changing lives study. Am. J. Public Health.

[57] Illgner T, Lad N (2022). Data to improve air quality environmental justice outcomes in South Chicago. Front Public Health.

[58] Hwa Jung K (2022). The effects of the historical practice of residential redlining in the United States on recent temporal trends of air pollution near New York city schools. Environ. Int..

[59] Adgate J L (2004). Outdoor, indoor, and personal exposure to VOCs in children. Environ. Health Perspect..

[60] Manson S (2023). National historical geographic information system: version 18.0 [Internet]. Minneapolis, MN: IPUMS.

[61] Kind A J H, Buckingham W R (2018). Making neighborhood-disadvantage metrics accessible — the neighborhood atlas. N. Engl. J. Med..

[62] Ferguson L, Taylor J, Davies M, Shrubsole C, Symonds P, Dimitroulopoulou S (2020). Exposure to indoor air pollution across socio-economic groups in high-income countries: a scoping review of the literature and a modelling methodology. Environ. Int..

[63] Longman J M, Passey M E (2013). Children, smoking households and exposure to second-hand smoke in the home in rural Australia: analysis of a national cross-sectional survey. BMJ Open.

[64] Casey J A (2015). Predictors of indoor radon concentrations in pennsylvania, 1989–2013. Environ. Health Perspect..

[65] Shiue I (2015). Correlations of indoor second-hand smoking, household smoking rules, regional deprivation and children mental health: Scottish health survey, 2013. Environ Sci. Pollut. Res..

[66] Kuntz B, Lampert T (2016). Social disparities in parental smoking and young children’s exposure to secondhand smoke at home: a time-trend analysis of repeated cross-sectional data from the German KiGGS study between 2003-2006 and 2009-2012. BMC Public Health.

[67] Office of the Assistant Secretary for Planning and Evaluation (2024). Poverty Guidelines [Internet].

[68] Shrubsole C, Taylor J, Das P, Hamilton I G, Oikonomou E, Davies M (2016). Impacts of energy efficiency retrofitting measures on indoor PM _2.5_ concentrations across different income groups in England: a modelling study. Adv. Build Energy Res..

[69] Ferguson L (2021). Systemic inequalities in indoor air pollution exposure in London, UK. Build Cities..

[70] Russo E T (2015). Comparison of indoor air quality in smoke-permitted and smoke-free multiunit housing: findings from the boston housing authority. Nicotine Tob Res..

[71] Lozano P, Homan S (2021). Disparities in smoking behavior by race/ethnicity in 10 diverse communities in Chicago: findings from sinai community health survey 2.0. J Immigr Minor Health.

[72] Hansel N N (2008). A longitudinal study of indoor nitrogen dioxide levels and respiratory symptoms in inner-city children with asthma. Environ. Health Perspect..

[73] Rogalsky D K, Mendola P, Metts T A, Martin W J (2014). Estimating the number of low-income Americans exposed to household air pollution from burning solid fuels. Environ. Health Perspect..

[74] Zhao H, Chan W R, Delp W W, Tang H, Walker I S, Singer B C (2020). Factors impacting range hood Use in California Houses and low-income apartments. Int. J. Environ. Res. Public Health.

[75] Memmott T, Konisky D, Carley S, Graff M (2020). Replication data for: sociodemographic disparities in energy insecurity among low-income households before and during the COVID-19 pandemic [Internet]. Harvard Dataverse.

[76] Center for Disease Control (2023). Ventilation in Buildings [Internet].

[77] Jones E R (2021). The effects of ventilation and filtration on indoor PM2.5 in office buildings in four countries. Build. Environ..

[78] Vu T V (2022). Assessing the contributions of outdoor and indoor sources to air quality in London homes of the SCAMP cohort. Build. Environ..

[79] Dockery D W (1993). An association between air pollution and mortality in six U.S. Cities. N Engl J Med..

